# A123 COMPARING SIZE MEASUREMENT OF SIMULATED COLORECTAL POLYPS WHEN USING A NOVEL VIRTUAL SCALE ENDOSCOPE, ENDOSCOPIC RULER OR FORCEPS: A BLINDED RANDOMIZED TRIAL

**DOI:** 10.1093/jcag/gwac036.123

**Published:** 2023-03-07

**Authors:** R Djinbachian, M Taghiakbari, C Haumesser, M Zarandi-Nowroozi, M Abou-Khalil, S Sidani, J Liu, B Panzini, D von Renteln

**Affiliations:** CHUM, Montreal, Canada

## Abstract

**Background:**

Accurate polyp size measurement is important for guideline conforming choice of polypectomy techniques and subsequent surveillance interval assignments. Some endoscopic tools (forceps or endoscopic rulers [ER]) exist to help with visual size estimation. A virtual scale endoscope (VSE) has been developed that allows superimposing a virtual measurement scale during live endoscopies.

**Purpose:**

Our aim was to evaluate the performance of VSE when compared to ER and forceps-based measurement.

**Method:**

We conducted a randomized trial to evaluate the relative accuracy of size measurement of simulated colorectal polyps when using: VSE, ER, and forceps. Six endoscopists performed 60 measurements randomized at a 1:1:1 ratio using each method. Primary outcome was relative accuracy in polyp size measurement. Secondary outcomes included misclassification of sizes at the 5, 10, and 20mm thresholds.

**Result(s):**

A total of 360 measurements were performed. The relative accuracy of biopsy forceps, ER, and VSE was 78.9% (95%CI=76.2-81.5), 78.4% (95%CI=76.0-80.8), and 82.7% (95%CI=80.8-84.8). VSE had significantly higher accuracy compared to biopsy forceps (p=0.02) and ER (p=0.006). VSE misclassified a lower percentage of polyps >5mm as ≤5mm (9.4%) compared to forceps (15.7%) and ER (20.9%). VSE misclassified a lower percentage of ≥20mm polyps as <20mm (8.3%) compared with forceps (66.7%) and ER (75.0%). 25.6%, 25.5%, and 22.5% of polyps ≥10mm were misclassified as <10mm with ER, forceps, and VSE, respectively.

**Conclusion(s):**

VSE had significantly higher relative accuracy in measuring polyps compared to ER or forceps assisted measurement. VSE improves correct classification of polyps at clinically important size thresholds.

**Please acknowledge all funding agencies by checking the applicable boxes below:**

None

**Disclosure of Interest:**

R. Djinbachian: None Declared, M. Taghiakbari: None Declared, C. Haumesser: None Declared, M. Zarandi-Nowroozi: None Declared, M. Abou-Khalil: None Declared, S. Sidani: None Declared, J. Liu: None Declared, B. Panzini: None Declared, D. von Renteln Grant / Research support from: Daniel von Renteln is supported by a “Fonds de Recherche du Québec Santé” (FRQS) career development award and has received research funding from ERBE, Ventage, Pendopharm, Fujifilm, and Pentax., Consultant of: Boston Scientific and Pendopharm,

